# Biodegradable Poly(lactic acid)-Based Blends as Intrinsic Self-Healing Matrices for Multifunctional and Eco-Sustainable Composites

**DOI:** 10.3390/molecules31060921

**Published:** 2026-03-10

**Authors:** Isacco Savioli, Laura Simonini, Daniele Rigotti, Alessandro Pegoretti, Andrea Dorigato

**Affiliations:** 1 Department of Industrial Engineering, University of Trento, Via Sommarive 9, 38121 Trento, Italy; isacco.savioli@studenti.unitn.it (I.S.); daniele.rigotti-1@unitn.it (D.R.); alessandro.pegoretti@unitn.it (A.P.); 2 National Interuniversity Consortium of Materials Science and Technology (INSTM), Via Giusti 9, 50121 Florence, Italy

**Keywords:** PLA, PBAT, blends, self-healing, composites, fracture toughness

## Abstract

In this work, compatibilized poly(lactic acid)/poly(butylene adipate-co-terephthalate) (PLA/PBAT) blends were developed and characterized, to be potentially utilized as biodegradable self-healing matrices for composite laminates. Blends containing 10, 20 and 30%wt of PBAT and 0.5 phr of an epoxy-based compatibilizer were prepared by melt compounding and hot pressing. Rheological measurements showed that moduli and complex viscosity generally increased with PBAT content, while maintaining viscosity levels suitable for conventional melt-processing operations. FT-IR and FESEM analyses confirmed the formation of an immiscible but well-compatibilized morphology, characterized by a homogeneous dispersion of PBAT domains within the PLA phase. Mechanical tests revealed a decrease in tensile modulus (up to 44%), strength (up to 45%) and fracture toughness (up to 40%) with a PBAT content up to 30%wt. Self-healing was evaluated by measuring the fracture toughness (K_IC_) recovery after thermal treatment at 140 °C. After healing, the blend containing 20%wt of PBAT exhibited a self-healing efficiency of 64% under impact conditions, which was attributed to the smoother fracture surface generated at an elevated strain rate that facilitated a more effective flow of the molten PBAT phase across the crack interface during healing. The formulation containing 20%wt of PBAT featured the best balance between mechanical performance and self-healing efficiency.

## 1. Introduction

Polymer composites are widely used as structural materials in transportation [1], aerospace [2], construction and energy [3,4] sectors thanks to their excellent combination of high specific strength, low density and corrosion resistance. However, despite these favourable characteristics, polymer composites are vulnerable to damage throughout their service life. Typical failure modes include matrix cracking [5,6], fibre/matrix debonding [7,8,9,10,11,12,13] and interlaminar fracture [14,15], which can compromise the load-bearing capacity of the structure and lead to catastrophic failure. However, damage inspection and reparation are often complex, time-consuming and costly. Many microcracks form and propagate within the polymer matrix or along interfaces, where they are not easily accessible to conventional non-destructive techniques. For these reasons, a significant interest has emerged in recent years for the development of polymer composites that can repair damage autonomously, known as self-healing composites, as a strategy to increase reliability, reduce maintenance and extend service lifetime [16].

Self-healing materials can generally be based either on extrinsic or intrinsic mechanisms. In materials with extrinsic self-healing capabilities, the healing agent is incorporated in microcapsules [17] or embedded in vascular networks [18]. The agent is released when cracks propagate through the material. While this approach effectively restores mechanical performance after damage, the system becomes inactive once the healing agent has been used, meaning that only one single healing event is possible. Furthermore, the microcapsules or networks may act as stress concentrators, causing defects in the matrix under load, or they may complicate the manufacturing process by increasing the viscosity of the matrix in the molten or liquid state [19]. By contrast, intrinsic self-healing materials rely on reversible interactions or polymer chain entanglement [20,21,22,23]. Consequently, intrinsic systems can undergo multiple healing cycles. Among the various intrinsic repair approaches, self-healing achieved by the incorporation of a thermoplastic polymer that melts (or softens) and flows into damaged areas has proven to be particularly promising. In this strategy, the self-healing agent is physically blended into a polymer matrix (both thermoplastic and thermosetting). When the material is heated above the melting (or the softening) temperature of the healing agent, it can penetrate and fill the cracks, allowing it to close and enabling mechanical recovery. The most common example is the repair of thermosetting matrices, such as epoxy resins, by the dispersion of polycaprolactone (PCL) particles [24,25]. More recent examples refer to the repair of thermoplastic matrices. For instance, Perin et al. [26] introduced a cyclic olefin copolymer (COC) healing agent into a polyamide 6 (PA6) matrix, achieving 35% self-healing efficiency in terms of fracture toughness recovery. Similarly, they introduced polycaprolactone (PCL) into PA6 with a 52% recovery of the fracture toughness [27]. Xu et al. [28] investigated polyurethane (TPU)/polycaprolactone (PCL) blends for self-healing application in order to achieve full recovery of the tensile properties with repeatable healing performance. The concept of intrinsic self-healing can be extended to biopolymers, driven by the growing demand for sustainable and environmentally friendly materials [29]. In this context, the integration of intrinsic self-healing biodegradable matrices with natural fibre reinforcements represents a highly attractive strategy for the development of advanced fully biodegradable composite materials [30]. While natural fibres such as flax, hemp and kenaf are increasingly employed to achieve lightweight and mechanically efficient composites, their susceptibility to damage and microcrack formation remains a critical limitation [31]. However, despite the growing interest in both self-healing polymers and biodegradable composites, their combined use remains largely unexplored. In this framework, polylactic acid (PLA) emerges as one of the most promising biopolymeric matrices [32,33,34,35,36,37,38]. Thanks to its renewable origin, biodegradability and good mechanical strength, it is used extensively in packaging [39] biomedical devices [40] and for structural components in lightweight applications [31]. However, PLA is brittle; therefore, a crack can form and propagate rapidly throughout the material, resulting in sudden and catastrophic failure [41]. One possible solution is to blend PLA with a thermoplastic biopolymer, such as poly(butylene adipate-co-terephthalate) (PBAT), which would make PLA tougher and self-healable [42,43]. PBAT is a biodegradable aliphatic–aromatic copolyester [44], and its rather low melting point (115–120 °C) makes it particularly promising in intrinsic self-healing applications, as it can melt and flow into cracks when heated to moderate temperatures. Despite its promising characteristics, the use of PBAT as a self-healing agent in PLA blends has not yet been investigated. Conversely, PLA/PBAT blends have been investigated as shape memory materials. For example, a previous work by Bianchi et al. [45] showed that PBAT domains in PLA/PBAT blends improved ductility and promoted shape memory capabilities. However, immiscibility and low interfacial adhesion were observed between the two polymers, which could be possibly improved by using compatibilizers such as epoxy-based chain extenders, which are able to react with the hydroxyl and carboxyl end groups of the polymer constituents and thus promoting the compatibilization [46]. For instance, Zakizadeh et al. [47] showed that the addition of an epoxy-based compatibilizer in PLA/PBAT blends significantly improved interfacial adhesion, resulting in enhanced mechanical performance and shape memory recovery.

On the basis of these considerations, the aim of this work was to develop biodegradable PLA/PBAT blends compatibilized with an epoxy-based chain extender, to be potentially utilized as novel self-healing matrices in fully biodegradable composite materials. To the best of the authors’ knowledge, no previous study has systematically addressed this investigation. These blends were characterized in terms of their rheological, morphological, and thermo-mechanical properties, paying particular attention to their fracture toughness both under quasi-static and impact conditions. The self-healing capability was evaluated by measuring the recovery of fracture toughness after a thermal healing treatment performed at 140 °C, i.e., above the melting point of the healing agent (PBAT).

## 2. Results and Discussion

### 2.1. Rheological Measurements

**Figure 1:**
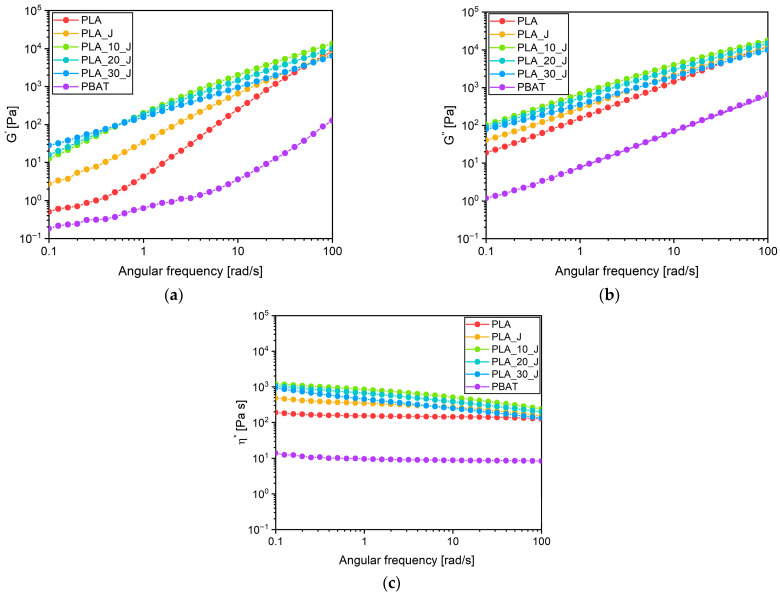
Dynamic rheological behaviour of the produced blends. Trends of (**a**) storage modulus, (**b**) loss modulus and (**c**) complex viscosity as a function of the angular frequency.

In Figure 1a–c, the results obtained from the dynamic rheological measurements on the prepared blends in terms of storage modulus (G′), loss modulus (G″), and complex viscosity (η*) are respectively reported.

The dynamic rheological properties of the blends highlight the influence of the compatibilizer and PBAT on the viscoelastic behaviour at the molten state of the resulting materials. The incorporation of the compatibilizer (J) increases the melt elasticity of PLA across the entire frequency range, as demonstrated by the increase in G′. This is consistent with its role as a chain extender and brancher. Indeed, it promotes the formation of a more entangled structure, which slows down chain relaxation and enhances the elastic response of the melt. The addition of PBAT further increases the G′ value, indicating that it possibly disperses as fine domains within the PLA matrix, thereby restricting its chain mobility in the molten state [48,49]. Notably, PLA_30_J exhibits higher G′ than PLA_10_J at frequencies up to 0.7 rad/s. However, at higher frequencies this trend reverses. Indeed, at low frequencies, a higher PBAT content promotes PLA/PBAT chain entanglements in the presence of the compatibilizer, thereby increasing the melt elasticity of the matrix and hindering its molecular relaxation. At higher frequencies, the influence of chain entanglements becomes less relevant due to the faster relaxation of the PBAT phase. This results in a decrease in G′ with increasing PBAT content, consistent with previous observations by Li et al. [50]. On the other hand, comparing the loss and storage moduli, it can be seen that G″ is greater than G′ across the entire frequency range for all the formulations, suggesting that liquid-like rheological behaviour prevails over a solid-like one. Furthermore, G″ slightly decreases with the PBAT amount; this trend was already observed by Bianchi et al. [45]. Regarding the complex viscosity, all formulations exhibit shear-thinning behaviour, which is more pronounced in the blends than in the neat polymers [50]. The η* of neat PLA is approximately one order of magnitude higher than that of PBAT, as shown in Figure 1c, and this indicates that PBAT can easily flow and disperse within the molten PLA matrix during healing. As expected, the incorporation of the compatibilizer slightly increases the viscosity of PLA, a trend that is consistent with the literature and with the role of J as chain extender [48]. Moreover, PLA viscosity is further enhanced by the introduction of 10%wt of PBAT. At this composition, the compatibilizer effectively acts as a chain extender, promoting chemical bonding between PBAT and PLA by the possible interaction with the carboxyl end groups of PLA on one side and the hydroxyl end groups of PBAT on the other. This further increases the viscosity compared to PLA_J. However, when the PBAT content is further increased to 20%wt and 30%wt, it could be hypothesized that the amount of compatibilizer used (i.e., 0.5 phr) could be insufficient to enable further bonding between PBAT and PLA phases. As a result, the free residual PBAT domains, which have a lower viscosity, lead to an overall (even if slight) reduction in the matrix’s viscosity [50]. However, all the compatibilized blends show melt viscosity values suitable for ensuring efficient processability under standard melt-processing conditions [51].

### 2.2. Chemical and Microstructural Characterization

**Figure 2:**
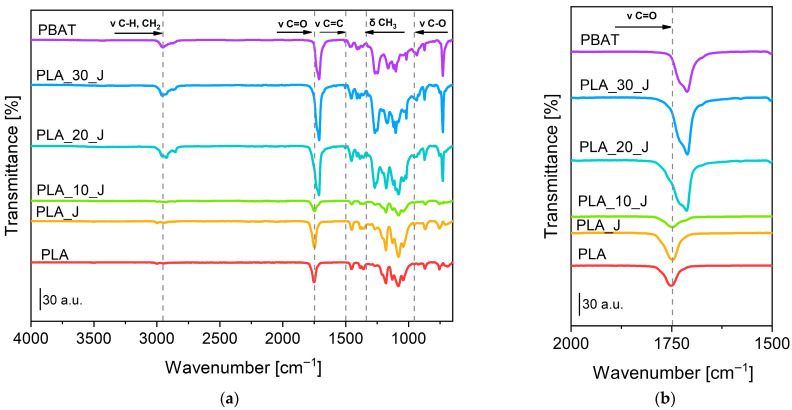
FT-IR spectra of neat PLA, neat PBAT and of the prepared blends, (**a**) from 4000 to 650 cm^−1^ and (**b**) enlargement from 2000 to 1500 cm^−1^.

In Figure 2a,b, the FT-IR spectra of the neat matrices and of the prepared blends are shown.

Neat PLA exhibits C-H stretching at around 2995–2945 cm^−1^, C=O stretching at 1750 cm^−1^ and CH_3_ bending at 1456 cm^−1^. In the region of 1300–1000 cm^−1^, it is possible to observe the stretching of -C-O- bond at approximately 1200–1080 cm^−1^, the ester groups at 1270 cm^−1^ and the asymmetric stretching of O-C at about 1080 cm^−1^ [52]. Between 1000 cm^−1^ and 800 cm^−1^ there are characteristic vibrations of the helical backbone with (CH_3_) rocking [53]. On the other hand, the PBAT spectrum shows an asymmetric stretching vibration of (-CH_2_-) at approximately 2956 cm^−1^, a more pronounced stretching vibration of the C=O group at 1711 cm^−1^ and stretching vibration of the C=C bond belonging to the benzene ring at 1507 cm^−1^. Peaks at 1268 cm^−1^, 1124 cm^−1^, 1099 cm^−1^ and 929 cm^−1^ correspond to (-C-O-) groups of the backbone connected to the benzene rings [54,55]. The spectra of the compatibilized PLA/PBAT blends show a combination of the characteristic bands of the two components, confirming the presence of these two polymers in the formulated blends. As the PBAT content increases, the C=O band progressively increases in intensity and shifts towards lower wavenumbers. This is consistent with the fact that the carbonyl groups present in PBAT absorb at lower wavenumbers (1711 cm^−1^) than those present in PLA phase (1750 cm^−1^). Moreover, the absence of any new bands besides those of neat PLA and PBAT, or significant chemical band shifts suggests that the PLA/PBAT system is probably immiscible. Furthermore, no additional peaks are detected and attributed to the presence of the compatibilizer, possibly due to its low concentration.

**Figure 3:**
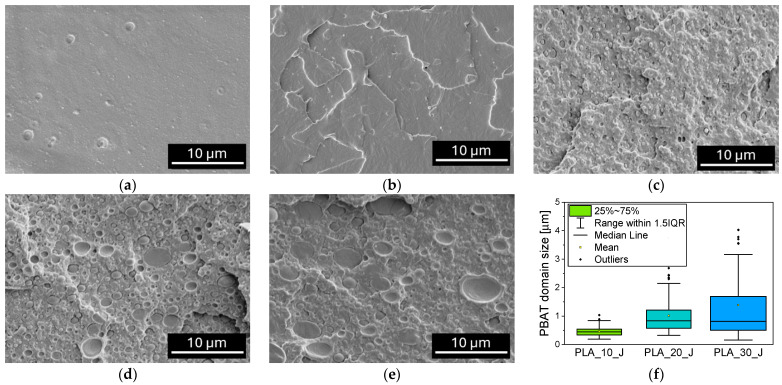
FESEM micrographs of the prepared blends, (**a**) PLA, (**b**) PLA_J, (**c**) PLA_10_J, (**d**) PLA_20_J, (**e**) PLA_30_J. (**f**) Size of PBAT domains in the blends.

Figure 3a–e show the FESEM micrographs of the prepared blends, while in Figure 3f a quantitative analysis of the PBAT domain size in these blends is reported.

Neat PLA (Figure 3a) exhibits a smooth fracture surface, characteristic of brittle polymers, while the addition of the compatibilizer creates a more ductile fracture morphology (Figure 3b). This is consistent with the role of the compatibilizer as a chain extender, which increases the molecular weight and entanglement density of PLA, thus altering the viscoelastic response of the matrix and promoting its localized plastic deformation during fracture [56]. In Figure 3c–e, the characteristic morphology of immiscible blends is shown, with PBAT appearing as spherical domains dispersed within the PLA matrix. These domains exhibit good interfacial adhesion with the PLA matrix, a property that can be attributed to the presence of the compatibilizer. As the PBAT content increases from 10 to 30%wt, the PBAT droplets progressively evolve in size, and a quantitative ANOVA analysis confirms this trend (Figure 3f). Specifically, PLA_10_J exhibits the smallest PBAT domains, with a median diameter of 0.44 µm and a narrow interquartile range (IQR = 0.20 µm). An increase in the characteristic domain size is observed for PLA_20_J, which shows a mean diameter of 0.83 µm and a broader dispersion (IQR = 0.64 µm). In PLA_30_J, the median diameter remains comparable (0.82 µm), but the distribution becomes markedly wider (IQR = 1.18 µm), indicating a substantial increase in statistical variability. The relatively narrow size distributions in PLA_10_J and, to a lesser extent, in PLA_20_J support the compatibilizing effect played by J at low PBAT concentrations, promoting the formation of finer and more uniformly dispersed domains within the PLA matrix [57]. In contrast, PLA_30_J is characterized by the coexistence of small and very large domains, indicating thus the onset of droplet coalescence and a reduced compatibilization efficiency at higher PBAT loadings (see also the viscosity trend shown in Figure 1c) [58].

### 2.3. Thermal Characterization

**Figure 4:**
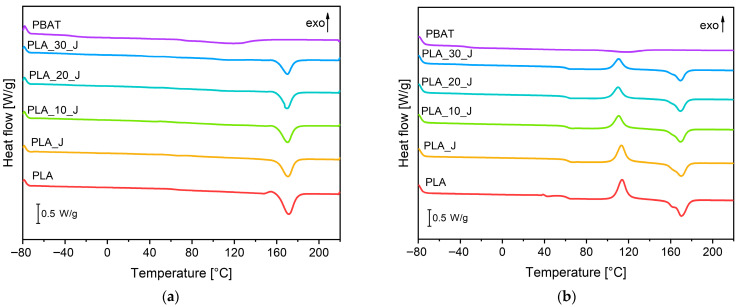
DSC thermograms of the produced blends: (**a**) first heating scan and (**b**) second heating scan.

Figure 4a,b show the DSC thermograms of the prepared blends collected during the heating scans (the cooling stage was not shown for brevity), while the most important results are summarized in Table 1.

During the first heating stage, the glass transition temperature of PLA is 64.4 °C and its melting temperature is 171.5 °C. The crystallinity of PLA is 39%, with no cold crystallization observed due to the annealing step performed at 100 °C after the hot pressing process [59]. A small exothermic peak precedes the melting of PLA, which corresponds to the reorganization of the α’-crystal of PLA into an α-crystal, as observed by Bianchi et al. [45]. The addition of the compatibilizer to PLA does not seem to significantly alter its thermal response. On the other end, PBAT exhibits a glass transition temperature of −34.3 °C and a melting temperature of 118.2 °C, with 27% degree of crystallinity. The progressive addition of PBAT induces a small reduction in the T_g_ of PLA, while no significant changes can be observed for the T_m_, further confirming the immiscibility of the PLA/PBAT system. Furthermore, PBAT introduction slightly reduces the crystallinity of PLA, that passes from 40% up to 35% with a PBAT amount of 30%wt. As Heidari et al. [58] have observed, the presence of well-compatibilized PBAT domains with high interfacial adhesion to the matrix restricts the mobility of PLA chains, thereby sligthly hindering the formation of crystals. In the second heating scan, the T_g_ of PLA is about 3 °C lower than that observed in the first scan, due to the removal of the thermal processing history and the relaxation of residual stresses [58], while the T_g_ of PBAT remains essentially unchanged. Furthermore, the T_m_ of neat PLA and PBAT are similar to those determined in the first scan. Instead, cold crystallization of the PLA occurs, reflecting the polymer’s latent crystallization potential, which is activated when a sufficient molecular motion is achieved [58]. This means that, regardless of the relative composition, the PLA matrix tested in the second heating stage is completely amorphous. This result highlights the crucial role of the annealing step performed at 100 °C during processing. In the absence of annealing, the PLA matrix would remain predominantly amorphous, compromizing thus the dimensional stability of the PLA matrix during the thermal healing process at 140 °C (well above the T_g_ of PLA) and thus the self-healing capability of the material. Possibly, X-ray diffraction (XRD) or polarized optical microscopy (POM) could help to clarify changes in the crystalline structure, and this aspect could be considered in a future development of this work, especially when the effect of multiple self-healing events will be explored. However, at a general level it can be concluded that the introduction of PBAT does not lead to substantial changes in the main thermal transitions of the PLA matrix, confirming the immiscible nature of the prepared blends.

**Figure 5:**
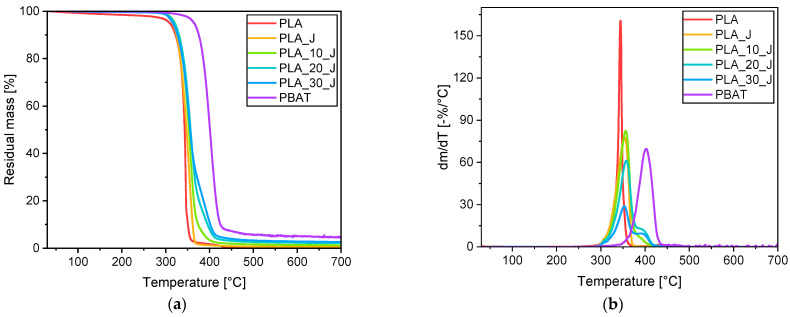
TGA tests on the prepared blends: trends of (**a**) residual mass and (**b**) mass loss derivative as a function of the temperature.

Figure 5a,b respectively show the TGA thermograms and the first derivative of the mass loss of the prepared blends, while the most important results are summarized in Table 2.

Both neat PLA and PBAT undergo thermal degradation in one single step. PLA loses 5% of its mass at 310 °C and shows maximum degradation rate at 345 °C. On the other hand, PBAT present a higher degradation resistance of PBAT, probably due to the presence of the benzene rings in its structure [60], as it loses 5% of its mass at 364 °C, while the maximum degradation rate occurs at 404 °C. Regarding the residual mass, PLA is completely degraded at 700 °C, leaving no solid residue, while PBAT present a m_700_ values of 4.6%, which indicates partial coking and carbonization, as already observed by Bianchi et al. [45]. The addition of the compatibilizer to PLA does not substantially alter its thermal degradation response. Regarding the blends, degradation occurs in two steps, respectively associated with the thermal degradation of PLA and PBAT phases, and this further demonstrates the immiscibility of the two polymers [45]. As a general conclusion, the thermal degradation stability of the blends, evaluated in terms of T_5%_, is slightly increased upon PBAT addition (about 10 °C). However, these temperatures are well above the service conditions in which these materials, if applied in biodegradable composites, could be typically used.

### 2.4. Mechanical Characterization

**Figure 6:**
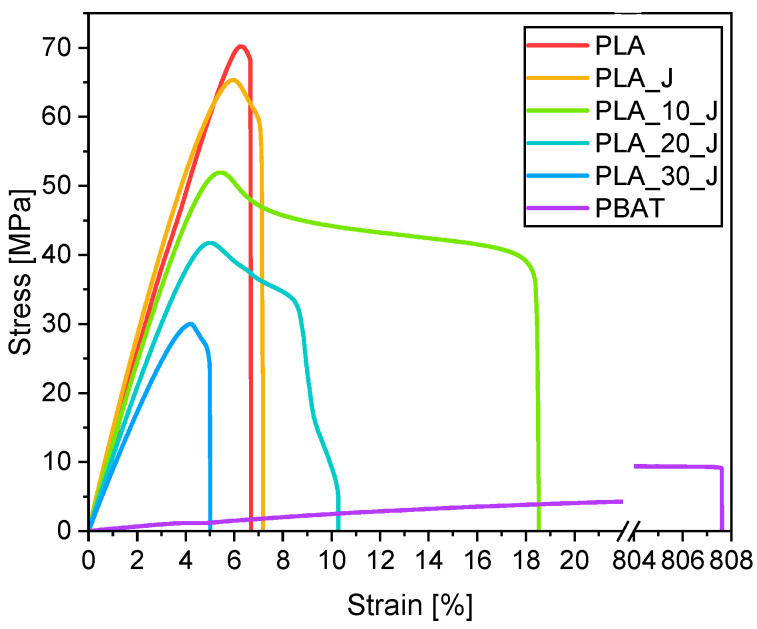
Representative stress–strain curves from quasi-static tensile tests on the prepared blends.

Figure 6 shows representative stress–strain curves from the tensile tests performed on the prepared blends, while Table 3 summarizes the main results collected in tensile and Vicat grade tests.

Neat PLA is characterized by an elastic modulus of 3.4 GPa, a tensile strength of 69.4 MPa and an elongation at break of 6.5%, and these values are consistent with those reported in the literature [41]. By contrast, neat PBAT exhibits an elastic modulus of 0.1 GPa, a tensile strength of 9.8 MPa and an elongation at break of approximately 900%, consistent with the literature data [45]. The addition of the compatibilizer to PLA does not significantly alter its tensile properties. On the other hand, the addition of PBAT generally decreases the elastic modulus and strength of PLA, while increasing the elongation at break up to a PBAT content of 20%wt. Notably, PLA_30_J exhibits a 44% decrease in elastic modulus and a 45% decrease in tensile strength, coupled with a 26% decrease in elongation at break compared to neat PLA. This reduction in elongation at break at elevated PBAT contents could be due to the immiscible nature of the blends and the formation of a morphology characterized by large and irregularly shaped PBAT domains within the PLA phase (see FESEM micrographs in Figure 3e) [45]. These results could also highlight that an increase in the compatibilizer fraction would likely have improved the miscibility of the blends with 20%wt and 30%wt PBAT, leading to enhanced mechanical properties at break.

PLA exhibits the highest VST (i.e., 155 °C), indicating an excellent dimensional stability, typical of an annealed PLA [61,62], while PBAT has a VST of 76 °C, due to its intrinsically low T_g_ and stiffness. As the amount of PBAT increases in the blends, the VST decreases progressively, albeit not significantly up to a PBAT amount of 20%wt. Nevertheless, the VST values remain relatively high even at a PBAT loading of 30%wt, confirming that the compatibilized PLA/PBAT systems still retain suitable thermo-mechanical stability for being utilized as matrices of fully biodegradable composites [61].

The results reported in Table 4 describe the fracture behaviour of the prepared blends both in quasi-static and impact mode, and the trends of the K_IC_ and G_IC_ as a function of the PBAT content are summarized.

**Figure 7:**
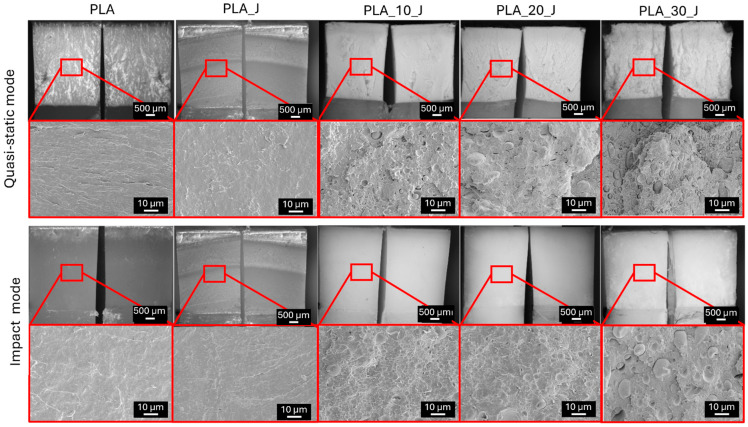
FESEM micrographs of the fracture surface of the SENB specimens tested in quasi-static and impact mode.

In quasi-static mode, the K_IC_ of neat PLA is 3.95 MPa/√m, which is consistent with the values reported in the literature for an annealed PLA [57]. Interestingly, the addition of the compatibilizer slightly reduces the K_IC_ of 19% with respect to neat PLA. A previous work by Altinbay et al. [57] has shown that epoxy-based chain extenders (like J) modify the molecular structure and rheological behaviour of PLA-based systems, by reacting with polymer chain ends, leading to chain extension and branching and by enhancing intermolecular cohesion and interfacial adhesion between polymer phases, rather than significantly altering the crystalline content. Indeed, in the present system, where crystallinity and elastic modulus of the PLA are not significantly altered by J introduction, the observed reduction in K_IC_ can therefore be attributed to changes in the local viscoelastic response and energy dissipation mechanisms near the crack tip caused by the chain extension. By increasing the PBAT content, K_IC_ is progressively reduced, with an approximate 40% decrease at 30%wt PBAT. This reduction can be mainly ascribed to the lower load-bearing capability of the blends, due to the partial immiscibility and the intrinsically limited mechanical properties of PBAT. Under quasi-static conditions, the PBAT domains therefore act primarily as mechanical discontinuities that facilitate crack initiation, rather than as effective toughening elements [63]. It is worth noting that this effect concerns the onset of unstable crack growth, while the subsequent crack propagation can still involve energy-dissipating mechanisms, as suggested by the fracture surface morphology and by the nearly constant G_IC_ values [64]. Conversely, the K_IC_ values in impact mode show contrasting trends. While neat PLA has a lower K_IC_ value (3.19 MPa/√m) than that in quasi-static mode, the PLA/PBAT blends have K_IC_ values higher than those measured in quasi-static conditions. One possible reason is related to the higher deformation rate, which modifies the viscoelastic response of the blends and leads to higher peak loads during testing. Since K_IC_ is directly related to the maximum applied load, this results in larger fracture toughness values in impact mode. This behaviour highlights the different nature of crack initiation in dynamic and quasi-static conditions, as observed by Perin et al. [27]. Moreover, the addition of increasing amounts of PBAT has no significant effect on the impact K_IC_ values. However, PLA_20_J shows a slight increase in K_IC_ (3.96 MPa/√m), suggesting a modest toughening effect that could be due to an optimized droplet-like morphology. Overall, the addition of PBAT alters the fracture toughness behaviour of PLA in a rate-dependent manner, reducing the resistance to crack propagation under quasi-static loading, while enabling limited toughening under impact conditions. The results obtained are further corroborated by the micrographs of the fracture surface of SENB specimens after testing in quasi-static and impact modes, shown in Figure 7.

The surface of the specimens tested in quasi-static conditions differs noticeably from that observed in impact mode. In quasi-static mode, the fracture surface of the blends is quite irregular and plastically deformed. Conversely, the surfaces of the specimens tested in impact mode are much smoother, with no evidence of plastic deformation, except for the PLA_30_J specimen, which exhibits some ductile surface deformation, likely due to its higher PBAT content. It is worth noting that a smoother fracture surface could facilitate the healing process, by enabling the healing agent to spread uniformly across the crack, whereas a rough, irregular surface could hinder this process [26,27].

### 2.5. Self-Healing Evaluation

Table 5 summarizes the fracture toughness values of the healed specimens (K_IC,H_), alongside the corresponding self-healing efficiency values, measured under quasi-static and impact mode.

**Figure 8:**
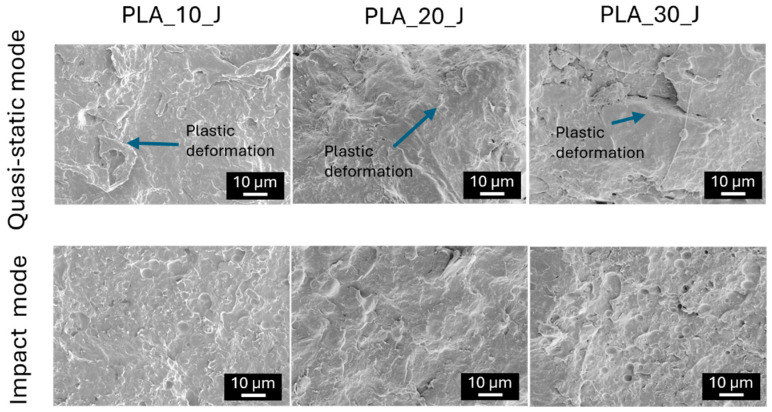
FESEM micrographs of the fracture surface of the healed SENB blend specimens after testing in quasi-static and impact mode.

By looking at the K_IC,H_ values, it is evident that the presence of PBAT is crucial for having a partial fracture toughness recovery. Neat PLA does not exhibit any recovery of K_IC_, and therefore its healing efficiency cannot be determined, and also the addition of 10%wt of PBAT is still insufficient to activate an effective self-healing mechanism. A clear change in healing behaviour is observed when the PBAT content increases to 20 and 30%wt, for which measurable fracture toughness recovery is obtained, reaching 4.7% for PLA_20_J and 9.8% for PLA_30_J. Self-healing is markedly more effective under impact conditions, with healing efficiencies of 36.1%, 63.7% and 49.5% for PLA_10_J, PLA_20_J and PLA_30_J, respectively. This enhanced repair performance in impact mode is probably attributed to higher testing speed, which produces smoother and less plastically deformed fracture surfaces (Figure 7), facilitating PBAT flow and spreading along the crack plane even at relatively low PBAT contents. In contrast, the application of quasi-static loading leads to highly deformed fracture surfaces, which hinder PBAT diffusion into the damaged regions. Notably, despite its higher PBAT content, PLA_30_J shows a lower healing efficiency than PLA_20_J in impact mode, likely due to the rougher fracture morphology observed in Figure 8, which limits crack-face alignment and PBAT flow [65].

The FESEM micrographs of the fracture surfaces of the SENB specimens after the healing process, tested again in quasi-static and in impact mode, are reported in Figure 8.

Regardless of the testing speed, the fracture surfaces after the healing process have a much smoother appearance, since PBAT domains are transformed into a thin film. This indicates that, upon healing, the PBAT phase melts and spreads along the crack plane, promoting crack closure and partial restoration of the fracture toughness. In quasi-static mode, however, the fracture surfaces still remain deformed and irregular, which limits the uniform flow of PBAT and hinders complete wetting of the damaged areas, resulting in a less effective repair. Conversely, in impact conditions, the smoother and more regular fracture morphology favours a more homogeneous redistribution of the PBAT film across the crack interface, thus explaining the higher healing efficiencies observed under impact loading.

Therefore, from the characterization performed, it can be concluded the blend containing 20%wt of PBAT represents the best balance between mechanical performance and self-healing efficiency. Therefore, this formulation will be investigated in the future for the fabrication of biodegradable composite laminates with self-healing capability.

## 3. Materials and Methods

### 3.1. Materials

**Figure 9:**
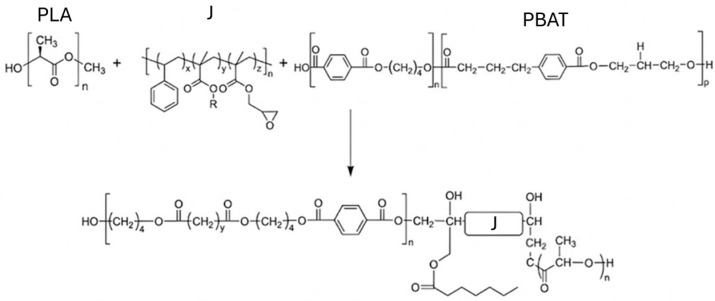
Schematic representation of epoxy ring-opening reactions of the compatibilizer with the terminal carboxyl (-COOH) and hydroxyl groups (-OH) of PLA and PBAT. In a preliminary investigation of our group on PLA/PBAT blends with a PBAT amount of 20%wt and 30%wt (not reported for brevity), it was clearly shown that the addition of the J compatibilizer led to an evident refinement of PBAT domains within the PLA matrix, markedly improving the interfacial adhesion and the resulting tensile mechanical properties. Therefore, in the present investigation only compatibilized PLA/PBAT blends have been considered.

The thermoplastic matrix was an Ingeo^®^ 4032D poly(lactic acid) (PLA), provided by NatureWorks LLC (Minnetonka, MN, USA) in form of granules of 3 mm. As reported in the datasheet, it had density of 1.24 g/cm^3^, molecular weight (M_w_) of 200,000 g/mol, melt flow index (MFI) of 7 g/10 min (210 °C, 2.16 kg) and melting temperature of 155–170 °C. The self-healing agent was a Technipol^®^ Bio 1160 poly(butylene adipate terephthalate) (PBAT), supplied by Sipol Spa (Mortara, Italy) in form of granules of 3 mm. According to the datasheet, it had density of 1.23 g/cm^3^, M_w_ of 50,000 g/mol, MFI of 20 g/10 min (160 °C, 2.16 kg) and melting temperature of 115 °C. The compatibilizer was an epoxy-based chain extender (indicated as J in the manuscript), sold under the name of Joncryl^®^ ADR 4468 and provided by BASF GmbH (Ludwigshafen am Rhein, Germany) in the form of 3 mm flakes. It is a copolymer of styrene, acrylic acid and glycidyl methacrylate, its epoxy functionality reacts with polycondensates (polyesters, polycarbonates and polyamides), enabling it to act as a chain extender and interfacial coupling agent. As reported in its datasheet, it had a density of 1.08 g/cm^3^ and glass transition temperature of 59 °C. The expected chemical reactions between PLA, PBAT and J are shown in Figure 9. The end groups of both PLA and PBAT are carboxyl (-COOH) and hydroxyl (-OH) groups. The compatibilizer can react with the polyesters by epoxy ring-opening reactions, which occur with the -COOH group of PLA on one side and the -OH group of PBAT on the other side. This can create a link between the two polyesters, with the compatibilizer backbone connecting the two chains.

### 3.2. Sample Preparation

Before processing, PLA, PBAT and J granules were dried in oven for 12 h at a temperature of 60 °C. Then, they were manually mixed at different relative amounts and subsequently compounded through a Thermo Haake Rheomix 600 internal mixer (Thermo Fisher Scientific Inc., Waltham, MA, USA) equipped with counter-rotating rotors, operating at 60 rpm for 6 min at a temperature of 180 °C. The obtained blends were then compression moulded in a Carver hot plate press (Carverpress Inc., Wabash, IN, USA) at 180 °C for 6 min, under an applied pressure of 3.4 MPa. In this way, square sheets of 120 × 120 × 2 mm^3^ were prepared. Thicker square sheets (120 × 120 × 5 mm^3^) were also prepared for the evaluation of the fracture toughness of these blends. The produced samples were then thermally annealed at 100 °C for 1 h, to enhance the crystallization of PLA [59] and to allow the thermal self-healing at 140 °C retaining the dimensional stability of the PLA matrix. The obtained blends are summarized in Table 6, where the corresponding fractions of each constituent are specified. A fixed amount of 0.5 phr of J was selected as the most suitable to compatibilize PLA/PBAT blends, as reported in the literature [47,66].

### 3.3. Experimental Techniques

#### 3.3.1. Rheological Properties

The dynamic rheological properties of the blends were analyzed by using a Discovery Hybrid Rheometer (DHR-2) (TA Instruments Inc., New Castle, DE, USA), adopting a plate–plate configuration with a plate diameter of 25 mm and by setting a plate gap of 1 mm. Tests were carried out at 180 °C by applying a strain amplitude of 1%. In this way, the trends of the storage modulus (G′), loss modulus (G″) and complex viscosity (η*) were investigated in an angular frequency (ω) range between 0.1 and 100 rad/s. One specimen was tested for each composition.

#### 3.3.2. Chemical and Microstructural Properties

The chemical properties of the blends were analyzed by using Fourier transform infrared spectroscopy (FT-IR) in attenuated total reflectance mode using a Perkin Elmer Spectrum One FTIR spectrometer (Perkin Elmer Inc., Shelton, CT, USA). Four scans in a wavenumber interval between 4000 and 650 cm^−1^ were performed for each sample, with a 4 cm^−1^ resolution. One specimen was tested for each composition.

The morphological features of the blends were observed on the cryo-fractured surfaces of both virgin and healed samples by using a field emission scanning electron microscope (FESEM) Zeiss Supra 40 (Carl Zeiss AG, Oberkochen, Germany), operating at an accelerating voltage of 3.5 kV. Prior to the analysis, the samples were coated with a layer of a platinum/palladium (Pt/Pd) alloy (80:20) with a thickness of about 5 nm. From the micrographs, the size of PBAT domains were measured by using ImageJ^®^ software (version 1.53a).

#### 3.3.3. Thermal Properties

Differential scanning calorimetry (DSC) tests were conducted through a Mettler DSC30 calorimeter (Mettler Toledo Inc., Columbus, OH, USA) under a nitrogen flow of 100 mL/min, in a temperature range from −80 °C to 220 °C at a heating/cooling rate of 10 °C/min. All samples were subjected to a first heating scan, a cooling scan and a second heating scan. In this way, the thermal characteristics of both PLA and PBAT phases in the blends were analyzed, i.e., their glass transition temperature (T_g_), melting temperature (T_m_), melting enthalpy (ΔH_m_), cold crystallization temperature (T_cc_) and cold crystallization enthalpy (ΔH_cc_). Equation (1) was used to calculate the degree of crystallinity of PLA and PBAT phases (X) in the blends,

(1) $$X(\%)=\frac{{∆H}_{m}-{∆H}_{cc}}{{∆H}_{m}^{0}\times w}\times 100$$

where ${∆H}_{m}$ is the measured melting enthalpy of PLA (or PBAT), ${∆H}_{cc}$ is the measured cold crystallization enthalpy of PLA (or PBAT), and ${∆H}_{m}^{0}$ is the melting enthalpy of the 100% crystalline PLA (93.7 J/g [41]) or PBAT (114 J/g [45]), while w is the weight fraction of PLA (or PBAT) in the blends. One specimen was tested for each composition.

Thermogravimetric analysis (TGA) of the blends was carried out by using a Mettler TG50 thermobalance (Mettler Toledo Inc., Columbus, OH, USA) under a nitrogen flow of 100 mL/min in a temperature interval from 30 to 700 °C at a heating rate of 10 °C/min. The tests allowed us to measure the temperature associated with a mass loss of 5%wt (T_5%_) and the maximum degradation temperature (T_d_) of PLA and PBAT, which corresponds to the peak of the first derivative of the thermogravimetric curve (DTG). Moreover, the residual mass after the test (m_700_) was taken. One specimen was tested for each composition.

#### 3.3.4. Mechanical Properties

Quasi-static tensile properties of the blends were analyzed using an Instron^®^ 5969 universal testing machine (Instron Inc., Norwood, MA, USA) equipped with a load cell of 10 kN. Dumbbell specimens type 1BA were tested according to the ISO 527 standard [67] at room temperature. The elastic modulus (E) was measured at a crosshead speed of 0.25 mm/min, imposing a maximum axial deformation level of 1%, the strain was recorded using a dynamic extensometer Instron^®^ model 2620-601 (gauge length of 12.5 mm). According to the ISO 527 standard, E was determined as a secant value between deformation levels of 0.05% and 0.25%. Tensile properties at break (i.e., ultimate tensile stress (UTS) and elongation at break (ϵ_b_)) were evaluated at a crosshead speed of 5 mm/min, without using the extensometer, testing at least five specimens for each composition.

The Vicat softening temperature (VST) of the samples was determined according to the ASTM D1525 standard [68]. Tests were conducted at a heating rate of 120 °C/h using an HDT-Vicat tester model MP/3 (ATS Faar Industries Srl, Milano, Italy), applying a load of 10 N to rectangular specimens of 10 × 10 × 5 mm^3^. Three specimens were tested for each composition.

The fracture toughness of the blends was evaluated in quasi-static mode according to ASTM D5045 standard [69]. The tests were carried out on Single Edge Notched Bending (SENB) specimens, having nominal dimensions of 44 × 10 × 5 mm^3^, an initial notch length of 5 mm and a span length of 40 mm. Tests in quasi-static mode were performed in a three-point bending configuration by using an Instron^®^ 5969 universal testing machine with a crosshead speed of 10 mm/min. At least six specimens were tested for each composition. Tests in impact conditions were performed using a Charpy impact machine (CEAST, Darmstadt, Germany), according to ISO 17281 standard [70]. The load–displacement curves were recorded using a tup extensometer in the hammer. A striker mass equal to 0.5 kg, a starting angle of 60° and an impact speed of 1.5 m/s were utilized, and at least six specimens were tested for each composition. From the load–displacement curves deriving from both quasi-static and impact tests it was possible to determine the maximum load sustained by the samples (P_max_), while the critical stress intensity factor (K_IC_) was calculated according to Equation (2),

(2) $$K_{IC}=\frac{P_{max}}{B\sqrt{W}}\cdot f(x)$$

where B and W are respectively the thickness and the width of the specimen and f(x) is a finite value provided in the standard as function of (x = a/W), where a is the notch length. Furthermore, in accordance with ASTM D5045 standard, the critical strain energy release rate (G_IC_) values in quasi-static mode were obtained by integrating the load–displacement curves and evaluating the system compliance, according to the expression reported in Equation (3),

(3) $$G_{IC}=\frac{∆U}{BW\varphi }$$

where ΔU is the difference in the total energy absorbed by the sample and the energy absorbed in the indentation tests, and φ is a finite value and it is provided in the standard as the function of x = a/W.

#### 3.3.5. Evaluation of Self-Healing Efficiency

**Figure 10:**
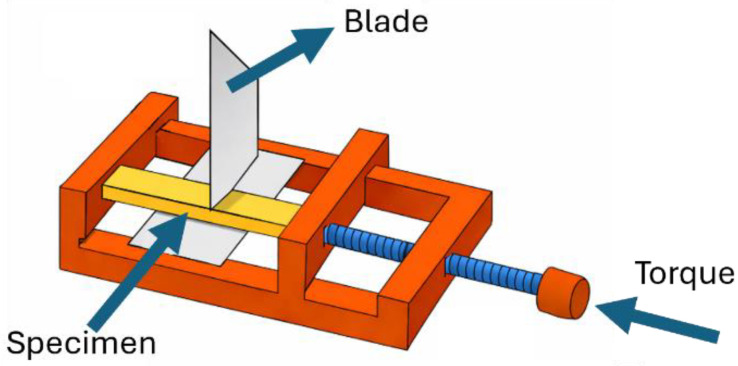
Schematic representation of the device used for the thermal healing process.

After being tested, the broken SENB specimens were placed in an iron vice under a pressure of 0.5 MPa (see Figure 10) and heated in an oven at 140 °C for 60 min. These parameters were selected after preliminary trials and on the basis of a previous paper by our group [15]. The selected temperature was higher than the melting temperature of PBAT (i.e., about 115–120 °C), in order to guarantee sufficient macromolecular mobility of the healing agent within the PLA matrix during the thermal healing process. On the other hand, preliminary results on these systems (not reported for brevity) showed that increasing the temperature beyond 140 °C did not lead to an improvement of the repair capability, and the materials would have thus been exposed to unnecessary harsh thermal treatment.

The healed specimens were then tested again in quasi-static (or impact) mode in order to measure the fracture toughness of the healed specimens (K_IC,H_). The healing efficiency (HE) was thus calculated by using the expression reported in Equation (4).

(4) $$HE=\frac{K_{IC,H}}{K_{IC}}$$

## 4. Conclusions

In this work, compatibilized poly(lactic acid)/poly(butylene adipate-co-terephthalate) (PLA/PBAT) blends were successfully developed and systematically characterized, to evaluate their potential as biodegradable self-healing matrices for structural composite laminates. Blends containing 10, 20 and 30%wt of PBAT and 0.5 phr of an epoxy-based compatibilizer were prepared by melt compounding and hot pressing. Their rheological, morphological, thermal and mechanical properties were investigated, with particular attention to the fracture toughness and thermal repair capability. Rheological analysis demonstrated that the incorporation of the compatibilizer significantly enhanced the melt elasticity of PLA, confirming its role as an effective chain extender and branching agent, while PBAT introduction further modified the viscoelastic response, increasing the melt elasticity due to the enhanced PLA/PBAT interfacial compatibility. Morphological observations highlighted the formation of immiscible but well-compatibilized blends, characterized by homogeneously dispersed PBAT domains with good interfacial adhesion to the PLA matrix. Thermo-gravimetric analyses showed that all the prepared blends maintained good thermal stability, with PBAT contributing to a slight improvement in the thermal degradation resistance. From a mechanical point of view, increasing PBAT content up to 30 wt% resulted in a reduction in stiffness (44%) and strength (45%), but enhanced elongation at break (26%) compared to neat PLA, due to the immiscibility and the intrinsically lower stiffness of the PBAT. The tests for the evaluation of the fracture toughness under quasi-static and impact conditions revealed that the addition of the chain-extending compatibilizer (J) slightly reduced the K_IC_ value compared to neat PLA, due to changes in the local viscoelastic response and energy dissipation mechanisms near the crack tip. By increasing the PBAT content, the value of K_IC_ of PLA progressively reduced, with an approximate 40% decrease in the quasi-static mode at 30%wt of PBAT. This decrease might be due to the fact that PBAT domains primarily acted as mechanical discontinuities that facilitated crack initiation rather than being effective toughening elements. Conversely, the K_IC_ values obtained in impact mode showed that increasing amounts of PBAT had no significant effect on the impact K_IC_ values. However, the blend containing 20%wt of PBAT exhibited a slight increase in K_IC_ in impact mode, indicating a modest toughening effect potentially resulting from an optimized droplet-like morphology. Self-healing was assessed by measuring the recovery of fracture toughness in SENB samples after they were heated to 140 °C. These tests revealed that PBAT was essential to obtain a partial fracture toughness recovery. While neat PLA and the blend containing 10%wt of PBAT showed negligible healing, a clear repair was observed for blends containing 20%wt and 30%wt of PBAT, with a self-healing efficiency of 64% achieved for the blend containing 20%wt of PBAT under impact. It was also shown that the repairability of these materials was strictly correlated with the strain rate applied during testing and the consequent formation of a flat and regular fracture surface morphology, which allowed effective diffusion of molten PBAT into the crack zone. The formulation with 20%wt of PBAT represented the best balance between mechanical performance and self-healing efficiency, and therefore it will be investigated in the future for the fabrication of biodegradable composite laminates with self-healing capability.

## Figures and Tables

**Table 1:** Results of the DSC tests on the prepared blends.

Samples	T_g,PLA_ (°C)	T_g,PBAT_ (°C)	T_cc,PLA_ (°C)	T_m,PLA_ (°C)	T_m,PBAT_ (°C)	∆H_cc,PLA_ (J/g)	∆H_m,PLA_ (J/g)	∆H_m,PBAT_ (J/g)	χ_PLA_ (%)	χ_PBAT_ (%)
First heating scan										
PLA	64.4	-	-	171.5	-	-	36.1	-	39.0	-
PLA_J	63.7	-	-	170.6	-	-	37.6	-	40.0	-
PLA_10_J	63.2	-	-	170.4	-	-	28.8	-	34.0	-
PLA_20_J	62.7	-	-	169.8	-	-	26.2	-	35.0	-
PLA_30_J	62.3	−36.7	-	170.1	-	-	23.0	-	35.0	-
PBAT	-	−34.3	-	-	118.2	-	-	31.2	-	27.0
Second heating scan										
PLA	61.8	-	113.8	170.2	-	37.9	38.1	-	≈0.0	-
PLA_J	61.9	-	113.4	170.0	-	32.6	32.8	-	≈0.0	-
PLA_10_J	61.4	-	110.6	169.5	-	28.1	28.1	-	≈0.0	-
PLA_20_J	60.6	-	110.1	169.3	-	24.5	24.5	-	≈0.0	-
PLA_30_J	60.4	−36.3	110.6	169.2	-	21.2	21.9	-	≈0.0	-
PBAT	-	−34.0	-	-	118.3	-	-	10.5	-	9.0

**Table 2:** Results of the TGA performed on the prepared blends.

Samples	T_5%_ (°C)	T_d,PLA_ (°C)	T_d,PBAT_ (°C)	m_700_ (%)
PLA	310	345	-	0.0
PLA_J	314	347	-	0.6
PLA_10_J	320	356	390	1.3
PLA_20_J	320	359	393	2.2
PLA_30_J	318	354	393	2.7
PBAT	364	-	404	4.6

**Table 3:** Quasi-static tensile properties and Vicat grade of the prepared blends.

Samples	E (GPa)	UTS (MPa)	ϵ_b_ (%)	VST (°C)
PLA	3.4 ± 0.2 ^a^	69.4 ± 2.9 ^a^	6.5 ± 0.6 ^a^	155.4 ± 3.3 ^a^
PLA_J	3.5 ± 0.5 ^a^	65.0 ± 0.7 ^a^	7.0 ± 0.9 ^a^	151.7 ± 1.4 ^a^
PLA_10_J	3.4 ± 0.6 ^a^	52.3 ± 0.9 ^b^	17.9 ± 2.4 ^b^	148.6 ± 2.4 ^a^
PLA_20_J	2.5 ± 0.1 ^b^	41.0 ± 0.5 ^c^	12.6 ± 4.5 ^c^	142.6 ± 2.5 ^a^
PLA_30_J	1.9 ± 0.1 ^c^	31.1 ± 0.9 ^d^	4.8 ± 0.4 ^a^	131.7 ± 12.1 ^b^
PBAT	0.1 ± 0.1 ^d^	9.8 ± 0.3 ^e^	896.1 ± 89.3 ^d^	75.9 ± 6.6 ^c^

Data are reported as mean ± standard deviation, the statistical significance of these results was assessed by one-way analysis of variance (ANOVA) followed by Tukey’s honestly significant difference (HSD) post hoc test (α = 0.05). The different superscript letters (a–e) denote statistically significant differences among groups (*p* < 0.05).

**Table 4:** Critical stress intensity factor (K_IC_) and critical strain energy release rate (G_IC_) of the prepared blends measured in quasi-static and impact modes.

	Quasi-Static Mode		Quasi-Static Mode
Samples	K_IC_ $(MPa/\sqrt{m}$)	G_IC_ (kJ/m^2^)	K_IC_ $(MPa/\sqrt{m}$)
PLA	3.95 ± 0.33 ^a^	8.15 ± 0.79 ^a^	3.95 ± 0.33 ^a^
PLA_J	3.19 ± 0.12 ^b^	4.76 ± 0.46 ^b^	3.19 ± 0.12 ^b^
PLA_10_J	2.98 ± 0.12 ^b^	7.84 ± 1.06 ^a^	2.98 ± 0.12 ^b^
PLA_20_J	2.93 ± 0.42 ^b^	9.99 ± 1.72 ^a^	2.93 ± 0.42 ^b^
PLA_30_J	2.35 ± 0.13 ^c^	8.69 ± 0.51 ^a^	2.35 ± 0.13 ^c^

Data are reported as mean ± standard deviation, the statistical significance of these results was assessed by one-way analysis of variance (ANOVA) followed by Tukey’s honestly significant difference (HSD) post-hoc test (α = 0.05). The different superscript letters (a–c) denote statistically significant differences among groups (*p* < 0.05).

**Table 5:** Critical stress intensity factor of healed blends (K_IC,H_) and self-healing efficiency values (HE) in quasi-static and impact mode.

	Quasi-Static Mode		Impact Mode	
Samples	K_IC,H_ $(MPa/\sqrt{m}$)	HE (%)	K_IC,H_ $(MPa/\sqrt{m}$)	HE (%)
PLA	-	-	-	-
PLA_J	-	-	-	-
PLA_10_J	-	-	1.27 ± 0.43	36.1 ± 7.1
PLA_20_J	0.14 ± 0.07	4.7 ± 1.7	1.89 ± 0.55	63.7 ± 10.0
PLA_30_J	0.22 ± 0.08	9.8 ± 3.6	1.38 ± 0.31	49.5 ± 10.4

**Table 6:** List of the prepared blends.

Samples	PLA (%wt)	PBAT (%wt)	J (phr *)
PLA	100	-	-
PLA_J	100	-	0.5
PLA_10_J	90	10	0.5
PLA_20_J	80	20	0.5
PLA_30_J	70	30	0.5
PBAT	-	100	-

* phr = parts per hundred resin, i.e., grams of J every 100 g of (PLA + PBAT).

## Data Availability

Data available on request.
